# High Glucose-Induced TRPC6 Channel Activation Decreases Glutamate Uptake in Rat Retinal Müller Cells

**DOI:** 10.3389/fphar.2019.01668

**Published:** 2020-02-14

**Authors:** Mingming Ma, Shuzhi Zhao, Jian Zhang, Tao Sun, Ying Fan, Zhi Zheng

**Affiliations:** ^1^ Department of Ophthalmology, Shanghai General Hospital, Shanghai, China; ^2^ National Clinical Research Center for Eye Diseases, Shanghai, China; ^3^ Shanghai Key Laboratory of Ocular Fundus Diseases, Shanghai Jiao Tong University, Shanghai, China; ^4^ Shanghai Engineering Center for Visual Science and Photomedicine, Shanghai General Hospital, Shanghai Jiao Tong University, Shanghai, China; ^5^ Shanghai Engineering Center for Precise Diagnosis and Treatment of Eye Diseases, Shanghai, China

**Keywords:** TRPC6 channel, diabetic retinopathy, Müller cells, glutamate uptake, reactive oxygen species

## Abstract

High glucose (HG) increases the production of reactive oxygen species (ROS), leading to decreased glutamate uptake in Müller cells. The transient receptor potential cation channel 6 (TRPC6) channel, an oxidative stress-sensitive Ca^2+^-permeable cationic channel, is readily detected in Müller cells and highly expressed under HG conditions. Yet, the effect of high glucose-induced TRPC6 channel activation in Müller cells is poorly understood. We hypothesized that TRPC6 channel activation mediates high glucose-induced decreases in Müller cell glutamate uptake. We found RNA interference (RNAi) of the TRPC6 channel abolished HG-induced decreases in glutamate uptake and cell death. HG also decreased the expression of the glutamate-aspartate transporter (GLAST), which is the most important transporter involved in glutamate uptake. The mRNA level of ciliary neurotrophic factor (CNTF) in rMC-1 cells and the release of CNTF in the culture media was decreased, but the mRNA levels of IL-6 and vascular endothelial growth factor (VEGF) were increased under HG conditions. After RNAi silencing in rMC-1 cells, the mRNA levels of CNTF increased, but IL-6 and VEGF levels decreased. Furthermore, TRPC6 knockdown (KD) decreased expression of glial fibrillary acidic protein (GFAP) and increased expression of Kir4.1, pointing to inhibition of HG-induced gliosis in rMC-1 cells. ROS and intracellular Ca^2+^ levels decreased after TRPC6 knockdown. Exposure to Hyp9 (10 μM), a highly selective TRPC6 channel agonist, can aggravate HG-induced pathological changes. Collectively, our results suggest TRPC6 channel activation is involved in HG-induced decreases in glutamate uptake in rMC-1 cells. These findings provide novel insights into the role of TRPC6 in HG-induced retinal neurovasculopathy and suggest TRPC6 is a promising target for drug development for diabetic retinopathy (DR).

## Introduction

Diabetic retinopathy (DR) is a leading cause of blindness in working-aged populations in developed countries and is traditionally regarded as a disorder of the retinal vessels. However, recent evidence demonstrates the pathogenesis of DR includes not only vascular changes, but also neuronal damage (Puro, 2002). It is becoming clear that changes in neuronal function and viability occur before blood-retina barrier (BRB) abnormalities in patients with diabetes and in diabetic animals (Martin et al., 2004; Barber et al., 1998). Unfortunately, the molecular and cellular mechanisms involved in the alteration and survival of retinal neurons in DR are poorly understood.

Retinas exposed to high glucose experience oxidative stress due to the increased production of reactive oxygen species (ROS), which is a key contributor to DR development (Lowell and Shulman, 2005). ROS target various mechanisms that contribute to DR, among which impairment of the glutamate-aspartate transporter (GLAST) in Müller cells has emerged as an important disease mechanism (Li and Puro, 2002). The GLAST is the only glial-type glutamate transporter in the retina (Harada et al., 1998). Therefore, glutamate clearance in the retina is mainly regulated by Müller cells. Glutamate is the major retinal excitatory neurotransmitter and is toxic when present at high concentrations, ultimately resulting in neurodegeneration (Sucher et al., 1997). Low extracellular glutamate levels in the retina are only possible with normally functioning Müller cells, which transport glutamate into cells through the GLAST on the cell membrane. It is clear diabetes-induced neuronal excitotoxicity damage is caused by excessive glutamate levels, which are typically the result of high levels of ROS in Müller cells (Puro, 2002; Jadhav et al., 2009). Some studies have shown reducing ROS production through the use of some antioxidants, such as superoxide dismutase (SOD), green tea, and taurine, can rescue the activity of the GLAST in Müller cells under HG conditions (Zeng et al., 2010; Silva et al., 2013). It is clear ROS generation is intracellular-Ca^2+^ dependent, and blocking Ca^2+^ influx can reduce the production of ROS (Yang et al., 2011).

The transient receptor potential canonical (TRPC) family (TRPC1–TRPC7) contributes to calcium influx, which is involved in the regulation of cell proliferation, differentiation, and various physiological functions (Pedersen et al., 2005). In particular, TRPC6 is one of the most extensively analyzed TRPC channels; its expression and function have been investigated in the retina, central nervous system, kidneys, and cardiovascular system (Onohara et al., 2006; Tai et al., 2008; Sachdeva et al., 2018). One study showed that TRPC6 expression levels were significantly higher in the retina of diabetic mice compared to normal mice, indicating an upregulation of TRPC6 transcripts under diabetic conditions, which was considered a response to the oxidative stress present under HG conditions (Sachdeva et al., 2018). At the cellular level, the TRPC6 channel has been identified in Müller cells (Da Silva et al., 2008), monocytes (Wuensch et al., 2010), platelets (Liu et al., 2008), podocytes (Yang et al., 2013), and hippocampal neurons (Liu et al., 2017). The ubiquitous distribution of TRPC6 indicates it may play roles in a wide range of physiological processes. TRPC6 activation contributes to the disease state, which is highlighted by the rescue of oxidative stress-induced dysfunction *via* TRPC6 channel inhibition (Liu et al., 2017). Inhibition of the TRPC6 channel in THP-1 cells can reduce the production of ROS under HG conditions (Li et al., 2019). However, functional studies investigating the channel have not been performed in Müller cells. Müller cells, spanning the whole retina from the inner limiting membrane to the outer limiting membrane, are the predominant macroglia and retinal-supporting cells. Their structural characteristics make Müller cells ideal cellular regulators of physiological and pathological responses in the retinal vasculature and neurons (Reichenbach and Bringmann, 2013). Thus, the effect and mechanism by which TRPC6 channels function in Müller cells under HG conditions need to be investigated.

Here, we hypothesized that the TRPC6 channel mediates decreased glutamate uptake in Müller cells, because TRPC6 channel activation increases intracellular Ca^2+^ concentrations, which is required for ROS generation. To address our hypothesis, we selected rat Müller cells (rMC-1), which are known to express TRPC6. The levels of TRPC6, GLAST, Kir 4.1, and GFAP in rMC-1 cells under HG conditions were analyzed by Western blotting. Glutamate uptake assays were used to determine the activity of the GLAST. Cell viability was assayed by CCK-8 assays. Cell apoptosis was evaluated by TUNEL assays and caspase-3 activity. Intracellular ROS levels were measured using the CM-H2DCFDA assay. A cell-based fluorometric assay was used to measure intracellular Ca^2+^ concentrations in rMC-1 cells loaded with a fluorescent Ca^2+^ indicator. The release of CNTF, IL-6, and VEGF from rMC-1 cells, and the mRNA levels of CNTF, IL-6, and VEGF in rMC-1 cells were evaluated by ELISA and qRT-PCR.

## Materials and Methods

### Cell Culture and TRPC6 siRNA Transfection

A rat Müller cell line (rMC-1) was purchased from Kerafast Inc. (Boston, MA). Cells were cultured in DMEM media plus 10% fetal bovine serum and 1% penicillin streptomycin and maintained at 37°C in a humidified 5% CO_2_ atmosphere. The medium was changed every 48 h. Cells cultured in 5 mmol/L glucose served as the control. Cells cultured in 25 mmol/L glucose represented the high glucose (HG) condition. rMC-1 cells grown in mannitol (20 mmol/L) served as the osmotic control. rMC-1 cells were used within passages 10 to 20.

To knock down the expression of TRPC6, we transfected the rMC-1 cells with siRNA, specifically targeting rat TRPC6 (sense, 5’- CAUACAUGUUUAAUGAUCAtt-3’, antisense, 5’- UGAUCAUUAAACAUGUAUGct-3’) or negative control (NC) siRNA (sense, 5’-UUCUCCGAACGUGUCACGUTT-30, and antisense, 50-ACGUGACACGUUCGGAGAATT-3’). The oligonucleotides were mixed with the Lipofectamine RNAiMAX Transfection Reagent (Life Technologies, Grand Island, NY, USA) according to the manufacturer’s protocol. After rMC-1 cells were grown to 50% confluency in different plates, cells were transfected. Media was replaced with HG media 7 h after transfection, and incubation continued for 48 h.

### Analysis of Cell Viability

The viability of rMC-1 cells was evaluated by CCK-8 assays using a microplate reader. Briefly, rMC-1 cells were seeded in 96-well plates at a density of 10 × 10^3^ cells/well and cultured for 48 h. Then, cells were incubated in DMEM in the presence or absence of high glucose combined with or without Hyp9 (5 and 10 μM) for 48 h. Subsequently, the cells were incubated with the CCK-8 reagent for 2 h at 37°C. Finally, the optical density at 490 nm was measured with a microplate reader (Bio-Tek, Inc., Winooski, VT, USA). Measurements of each of these conditions were repeated three times in the same plate.

### Enzyme-Linked Immunosorbent Assays (ELISAs)

Cells in 96-well plates were incubated in 50 μl culture media with or without HG for 48 h, and the culture supernatants were collected. The concentrations of CNTF, VEGF, and IL-6 were determined by enzyme-linked immunosorbent assays (ELISAs) kits (R&D Systems, Minneapolis, MN, USA), according to the manufacturer’s instructions.

### Quantitative Real-Time PCR Analysis (qRT-PCR)

Total RNA was extracted from cultured rMC-1 cells using TRIzol (Life Technologies Inc., Gaithersburg, USA) according to the manufacturer’s protocol. mRNA levels of CNTF, VEGF, and IL-6 were quantified by qRT-PCR. The sequences of the primers were as follows: CNTF (NCBI RefSeq NM_013166.1), F 5^’^- CGACTCCAAGAGAACCTCCA-3^’^ and R 5^’^- CCTTCAGTTGGGGTGAAATG-3^’^, IL-6 (NCBI RefSeq NM_ 012589.2), F 5^’^- TCCTACCCCAACTTCCAATGCTC-3^’^ and R 5^’^- TTGGATGGTCTTGGTCCTTAGCC-3^’^, VEGF (NCBI RefSeq NM_ 031836.3), F 5^’^- GCACCCACGACAGAAGG-3^’^ and R 5^’^- TGAACGCTCCAGGATTTA-3^’^, β-actin (NCBI RefSeq NM_ 031144.3), F 5^’^-CACTGCCGCATCCTCTTCCTC-3 and R 5^’^-TGCTGTCGCCTTCACCGTTCC-3^’^. β-actin served as the internal control. CNTF, VEGF, and IL-6 mRNA levels were normalized to β-actin levels, which served as the endogenous control to ensure equal starting amounts of cDNA. The control group was set as the calibrator with a value of 1, and the other groups were compared to this calibrator.

### Western Blot Analyses

rMC-1 cells, treated as described previously, were homogenized in lysis buffer (0.05 M Tris-HCl, pH 7.4, 0.15 M NaCl, 0.25% deoxycholic acid, 1% NP-40, 1 mM EDTA). The protein samples were separated by SDS-PAGE and electroblotted onto a polyvinylidene fluoride membrane (Millipore, Bedford, MA, USA). After being blocked in 4% skim milk, the membrane was incubated at 4°C overnight with rabbit anti-TRPC6 (1:500; Abcam, Shatin, Hong Kong), rabbit anti-GLAST (1:500; Abcam, Shatin, Hong Kong), rabbit anti-Kir4.1 (1:1,000; Abcam, Shatin, Hong Kong), and rabbit anti-GFAP (1:5,000; Abcam, Shatin, Hong Kong) antibodies. Anti-beta-actin (1:1,000 dilution, Cell Signaling Technology, Beverly, MA, US) was used as a loading control. The intensity of the bands was quantified by densitometry using Image J software (NIH, USA).

### Immunohistochemistry and Transferase-Mediated dUTP Nick-End Labeling Staining

To detect individual apoptotic cells, staining for transferase-mediated dUTP nick-end labeling staining (TUNEL) was carried out using a DeadEnd™ fluorometric TUNEL system kit (Promega, Madison, WI, US) according to the manufacturer’s instructions. Cell nuclei were counterstained with DAPI (1 μg/ml; Beyotime Institute of Biotechnology, Jiangsu, China). Samples were observed under a confocal laser scanning microscope (Zeiss LSM510; Carl Zeiss, Thornwood, NY). The numbers of total and TUNEL-positive nuclei were counted and analyzed using ImageJ/Imaris software.

### Intracellular Ca2+ Measurements

Intracellular Ca^2+^ levels were quantitatively determined by Fluo3/AM fluorescence. rMC-1 cells were incubated with the Ca^2+^ indicator (Fluo3-AM; 10 μM) for 0.5 h at 37°C in the dark, after which rMC-1 cells were washed twice to remove excess stain. Finally, the FLUo3-dependent fluorescence was determined by a FACScan at 488-nm excitation and 530-nm emission wavelengths.

### Measurement of Intracellular ROS Generation

rMC-1 cells prepared with different treatments as described above were seeded in 96-well plates and grown to 85% confluence. The generation of intracellular ROS was detected by the dichlorodihydrofluorescein (DCF) method using 5-(and-6)- carboxy-2’,7’-dichlorodihydrofluorescein diacetate (carboxy-H2DCFDA). The cells were gently washed with PBS and incubated with 3 μM carboxy-H2DCFDA in phenol red–free medium at 37°C for 30 min. Cells were washed with and maintained in SBS before images were captured using a cell imaging system. Image J software was used for analysis of fluorescent intensity.

### Glutamate Uptake Assay

rMC-1 cells were cultured in 24 well plates. The culture medium was removed, and the cells were incubated with medium containing mannitol and 5 mmol/L glucose or 25 mmol/L glucose for 24 h at 37°C. To determine the glutamate uptake capacity in rMC-1 cells after pretreatment, the cells were washed and incubated for 30 min in Kreb’s solution containing 119 mM NaCl, 2.5 mM CaCl_2_, 4.7 mM KCl, 1.0 mM MgCl_2_, and 1.2 mM KH_2_PO4. Then, rMC-1 cells were exposed to 10 mmol/l unlabeled glutamate and 0.5 μCi/ml of L-[2,3-^3^H] glutamate (New England Nuclear, Boston, MA, USA) for 60 min. L-glutamate uptake was terminated by rapid removal of the incubation buffer, and the cells were washed twice with ice-cold PBS. rMC-1 cells were subsequently lysed, and small aliquots were removed from each well for the determination of protein content. L-[2,3-^3^H]-glutamate content was determined by scintillation counting.

### Detection of Activated Caspase-3

The enzymatic activity of the caspase-3 class of proteases in rMC-1 cells was measured by a caspase-3 colorimetric assay kit (Promega, Madison, WI, USA) as previously described (Ma et al., 2018). The cleavage of a caspase-3 colorimetric substrate (DEVD-pNA) was measured at 405 nm using a microplate reader (Auto Bio Labtech Instruments, Co, Ltd, China).

### Data Analysis

All experiments were performed at least three times, and the values were presented as mean ± SD; statistical significance was assessed using two-tailed Student’s t test or one-way ANOVA, followed by Tukey’s *post hoc* test. *P* values are indicated with *, **, and ***, which correspond to values of 0.05, 0.01, and 0.001, respectively.

## Results

### Effects of the TRPC6 Channel on Müller Cell Glutamate Uptake

**Figure 1:**
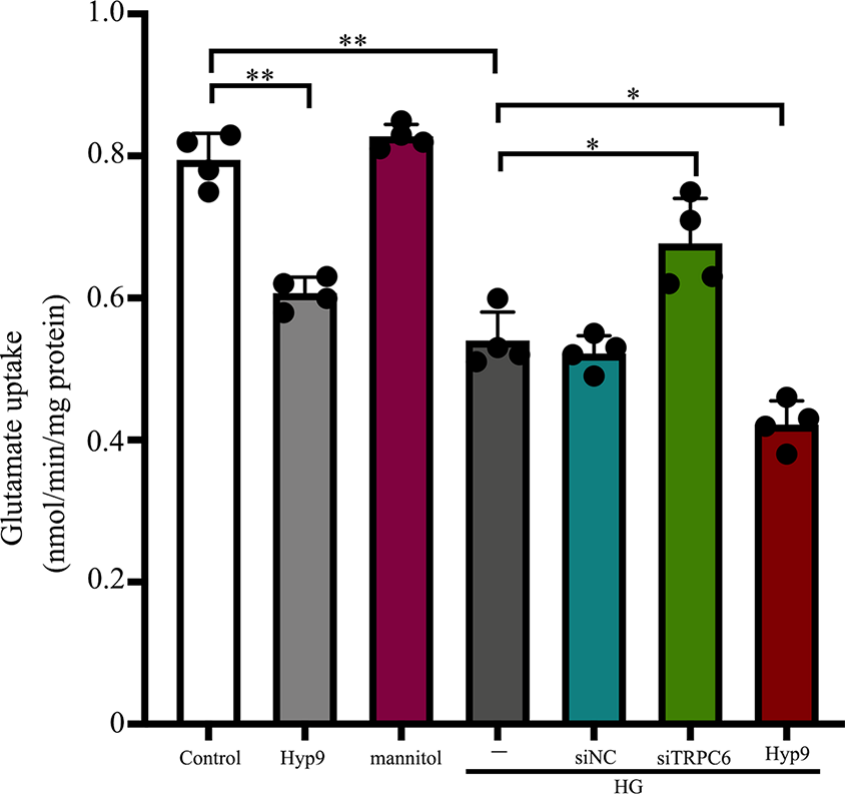
Effect of the TRPC6 channel on rMC-1 cell glutamate uptake under HG conditions. Glutamate uptake assays were performed in seven groups: control, Hyp9, mannitol, HG, HG + siNC, HG + siTRPC6, and HG + Hyp9 groups (n = 4). Data shown are mean ± SD, *p < 0.05, **p < 0.01.

The glutamate uptake assay showed that TRPC6 silencing (**Supplementary Figure 1**) could ameliorate glutamate uptake activity in retinal Müller cells under HG conditions. Exposure to 10 μM Hyp9 reduced glutamate uptake activity under HG conditions (**Figure 1**).

**Figure 2:**
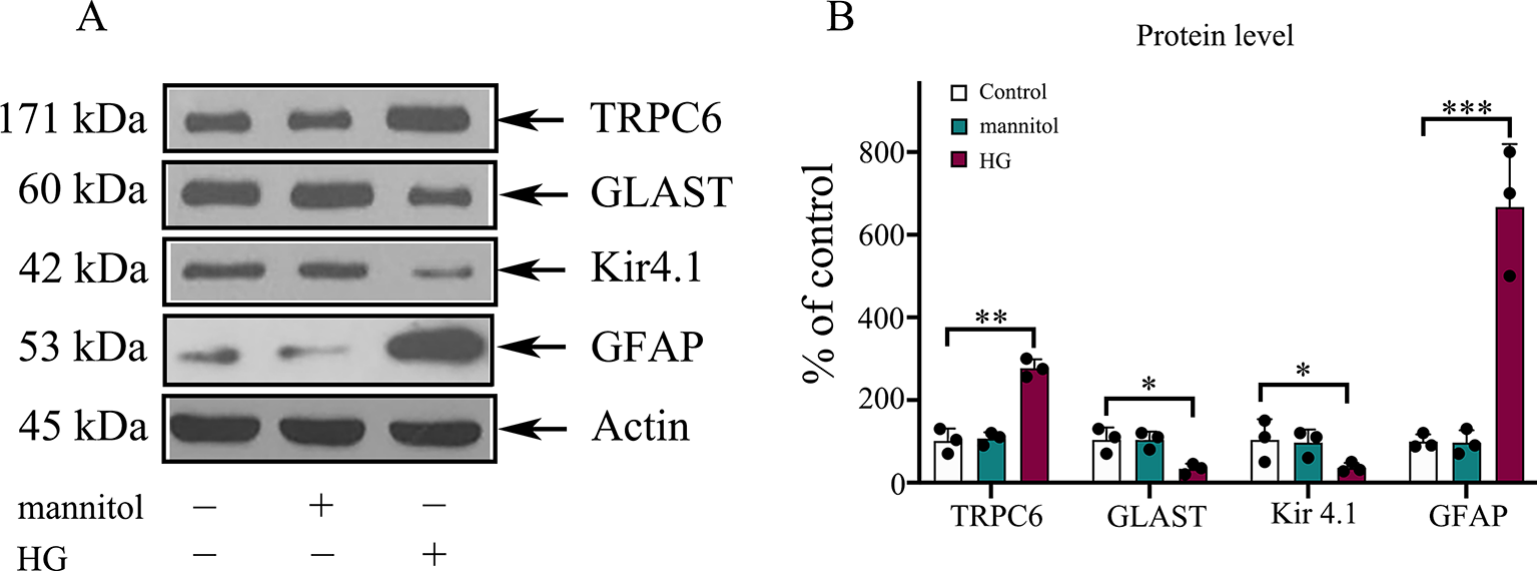
The protein levels of GLAST, Kir4.1, and GFAP in rMC-1 cells under HG conditions. **(A)** Western blotting was used for TRPC6, GLAST, Kir4.1, GFAP, and actin in rMC-1 cells under HG conditions. **(B)** Densitometric analyses of TRPC6, GLAST, Kir4.1, and GFAP protein levels were standardized against actin protein levels. Data are expressed as mean ± SD; n = 3 for each group; *p < 0.05, **p < 0.01, ***p < 0.001.

The synaptic glutamate levels in the retina are mainly regulated by the GLAST in Müller cells. DR caused downregulation of the inwardly rectifying potassium channel Kir4.1, possibly resulting in dysfunction of the GLAST (Napper et al., 1999). To determine the effect of TRPC6 channel activation on the expression of the GLAST and Kir 4.1, Western blot analysis was performed. We determined GLAST and Kir4.1 expression in Müller cells decreased under HG conditions (**Figure 2**). TRPC6 knockdown increased GLAST and Kir 4.1 expression significantly (**Figures 2** and **6**).

### Effect of the TRPC6 Channel on HG-Induced Müller Cell Viability and Apoptosis

**Figure 3:**
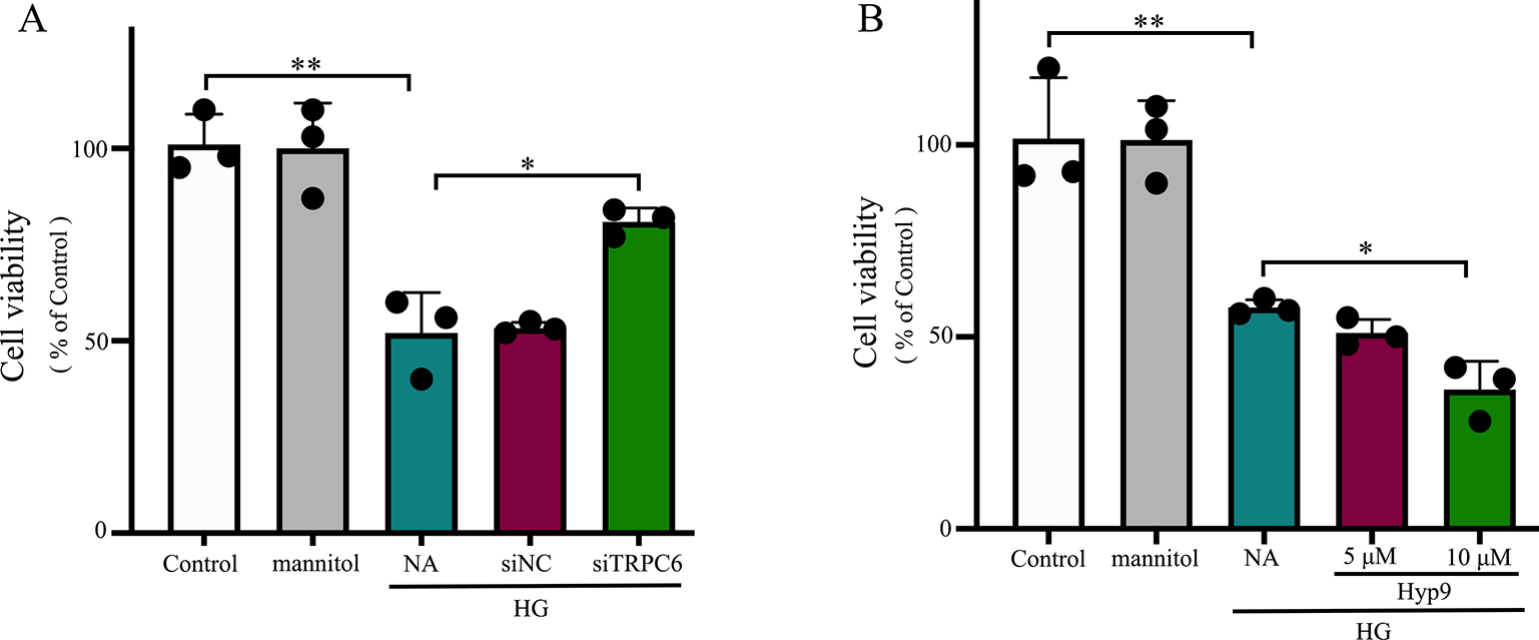
The effect of the TRPC6 channel on rMC-1 cell viability under HG conditions. **(A)** Decreasing TRPC6 expression in rMC-1 cell prevents HG-induced reductions in cell viability. **(B)** rMC-1 cells were treated with different concentrations (5 and 10 μM) of Hyp9. Activation of the TRPC6 channel by Hyp9 reduced cell viability, which was concentration dependent. The data are expressed as mean ± SD, as a percentage of control; n = 3 for each group; *p < 0.05, **p < 0.01.

To evaluate the influence of the TRPC6 channel on Müller cell viability, CCK-8 assays were used. Our results from three independent experiments are summarized in **Figure 3**. rMC-1 cells showed high cell viability under HG conditions after TRPC6 knockdown. Exposure to Hyp9 resulted in a concentration-dependent reduction in cell viability.

**Figure 4:**
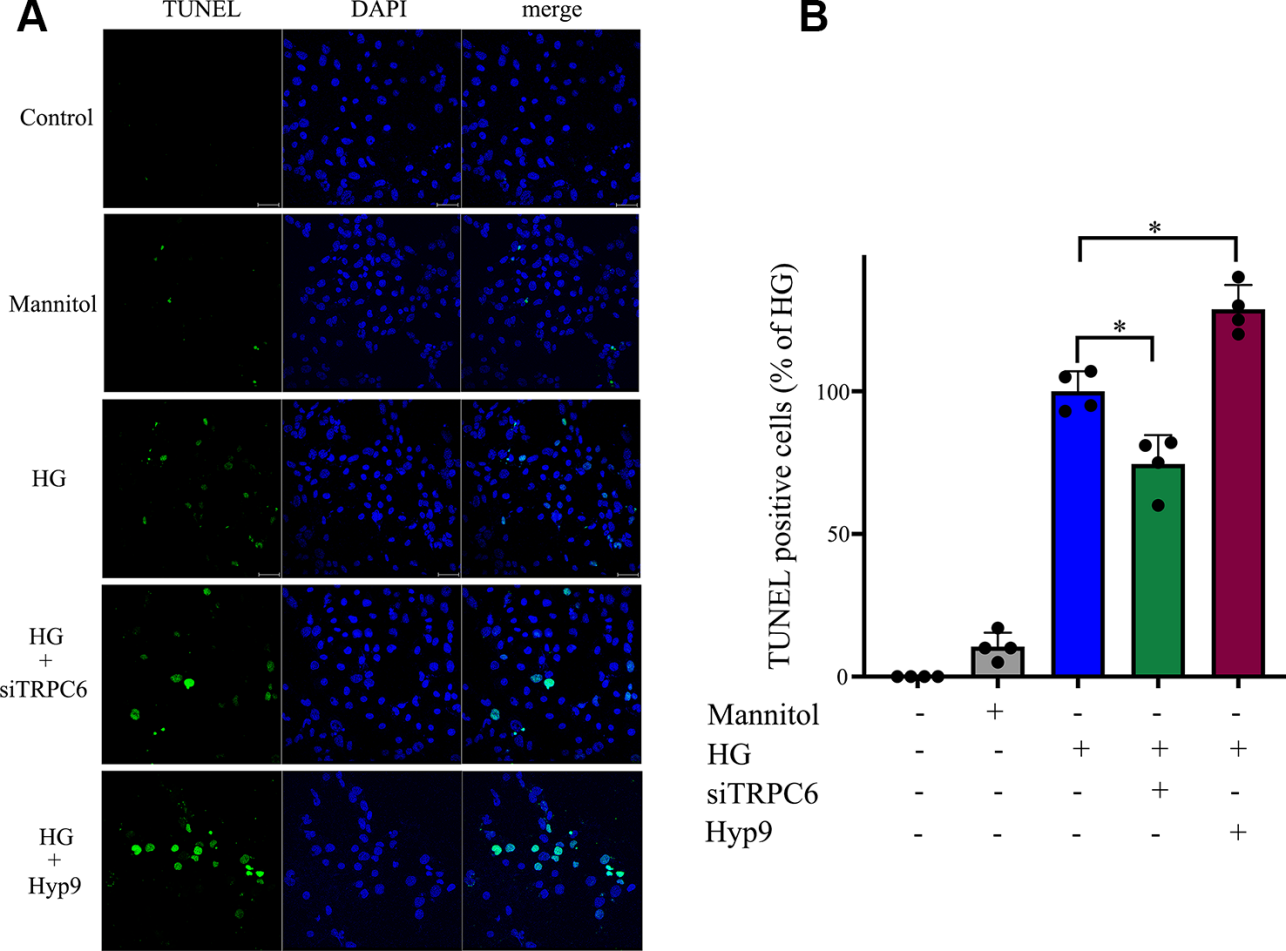
HG-induced rMC-1 cell apoptosis was inhibited by silencing TRPC6. TUNEL assays were used to detect cell apoptosis in rMC-1 cells. **(A)** The green staining cells under HG conditions were found. **(B)** The percentage of TUNEL positive cells compared with the HG group. The HG group was set as 100%. The data are expressed as mean ± SD; n = 3 for each group; *p < 0.05.

**Figure 5:**
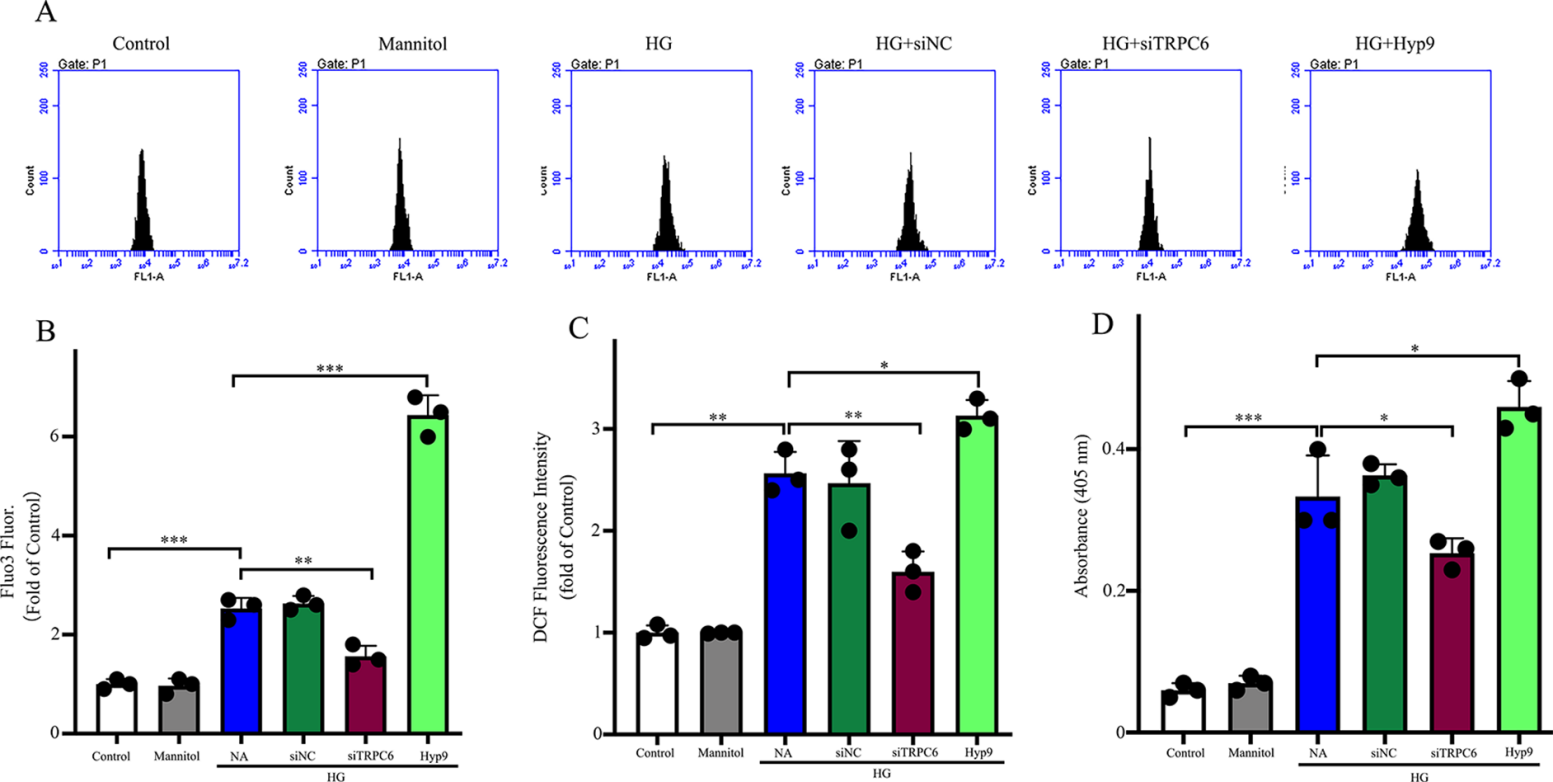
The effect of the TRPC6 channel on intracellular Ca^2+^ concentrations, ROS generation, and caspase-3 activity under HG conditions. **(A)** Representative images showing Fluo3 fluorescence as a function of cytosolic free Ca2+ in rMC-1 cells in different groups. **(B)** Densitometric analyses between different groups in **(A)**. **(C)** Generation of intracellular ROS was detected by the DCF method using carboxy-H2DCFDA. ROS production was inhibited by TRPC6 knockdown. **(D)** The high caspase-3 activities in rMC-1 cells under HG conditions were prevented by TRPC6 knockdown. The data are expressed as mean ± SD; n = 3 for each group; *p < 0.05, **p < 0.01, ***p < 0.001.

Consistent with previous reports, HG caused extensive cell apoptosis. In contrast, cells exposed to normal glucose concentrations or normal glucose plus mannitol (to exclude potential osmotic effects caused by excess glucose) underwent apoptosis at a much lower rate. HG caused significant cell apoptosis and increases in caspase-3 activity (**Figure 5D**). TRPC6 knockdown prevented cell apoptosis and decreased cellular caspase-3 activity (**Figures 4** and **5C**). These data indicate the TRPC6 channel plays a key role in HG-induced cell apoptosis.

### Effect of the TRPC6 Channel on ROS Generation in Müller Cells

To examine the effects of the TRPC6 channel on Ca^2+^ concentrations in Müller cells, cells were loaded with a fluorescent Ca^2+^ indicator dye, and the fluorescence ratio (F420/F480) was measured after excitation at 420 and 480 nm and served as a direct index of Ca^2+^ concentration. A shown in **Figure 5A**, HG increased the Ca^2+^ concentration in normal cells, but TRPC6 knockdown significantly decreased Ca^2+^ concentrations.

Previous studies have shown that Ca^2+^ signaling is required for ROS production (Duan et al., 2007). As ROS are involved in apoptosis and glutamate excitotoxicity (Flora et al., 2007), ROS production due to HG conditions was studied using the ROS-sensitive fluorescent dye CM-H_2_DCFDA. As shown in **Figure 5B**, HG promoted intracellular ROS accumulation in normal cells, but decreased concentrations in TRPC6 knockdown cells. Application of Hyp9 increased ROS accumulation under HG conditions in normal cells.

### Effect of the TRPC6 Channel on HG-Induced Müller Cell Gliosis

**Figure 6:**
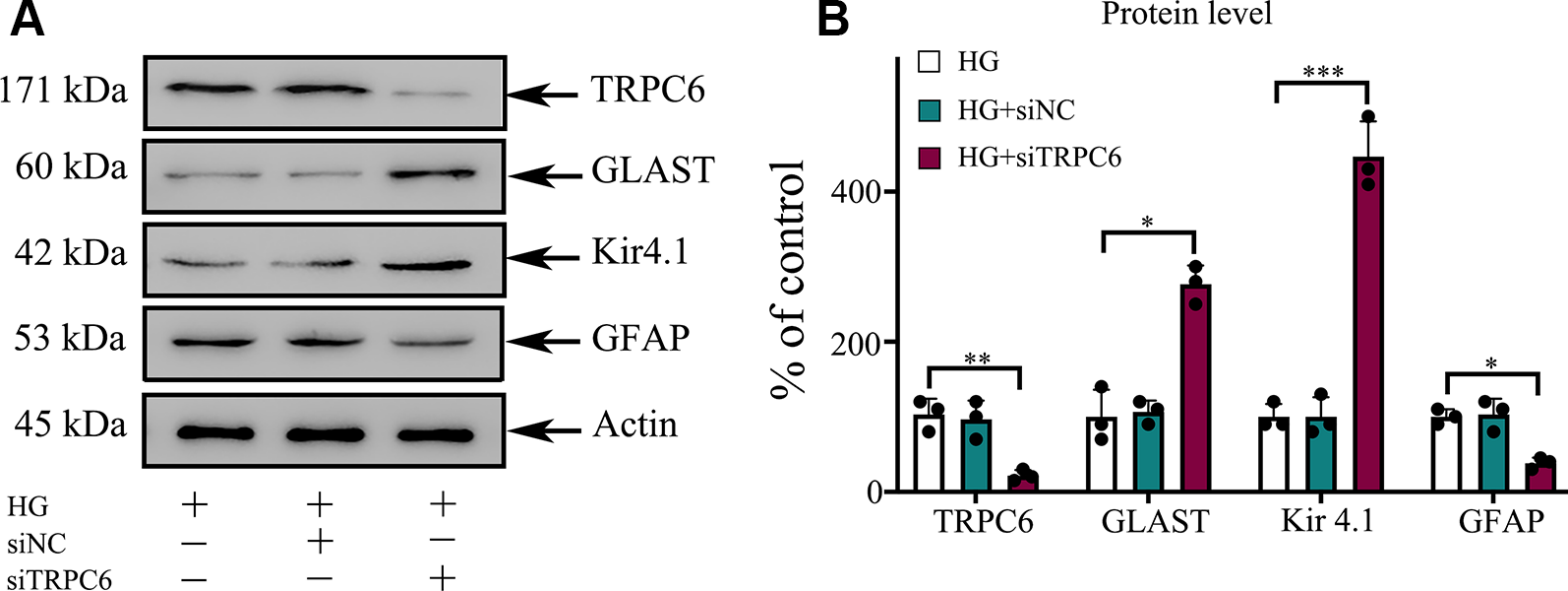
TRPC6 knockdown enhanced the protein content of GLAST and Kir4.1, but decreased the protein content of GFAP in rMC-1 cells under HG conditions. **(A)** Western blotting was used for TRPC6, GLAST, Kir4.1, GFAP, and actin in rMC-1 cells. **(B)** Densitometric analyses of TRPC6, GLAST, Kir4.1, and GFAP protein levels were standardized against actin protein levels. The data are expressed as mean ± SD, with the mean values for control set at 100%; n = 3 for each group; *p < 0.05, **p < 0.01, ***p < 0.001.

The gliosis of Müller cells is characterized by an upregulation of the immunoreactivity against intermediate filament constituents vimentin and GFAP. GFAP protein was used as a key marker of Müller cell gliosis under HG conditions. The GFAP protein level in the HG group was higher than in the normal control group (**Figure 2**). TRPC6 knockdown significantly decreased the protein levels of GFAP under HG conditions (**Figure 6**).

### Effect of the TRPC6 Channel on CNTF, IL-6, and VEGF Levels in Müller Cells and Supernatants

**Figure 7:**
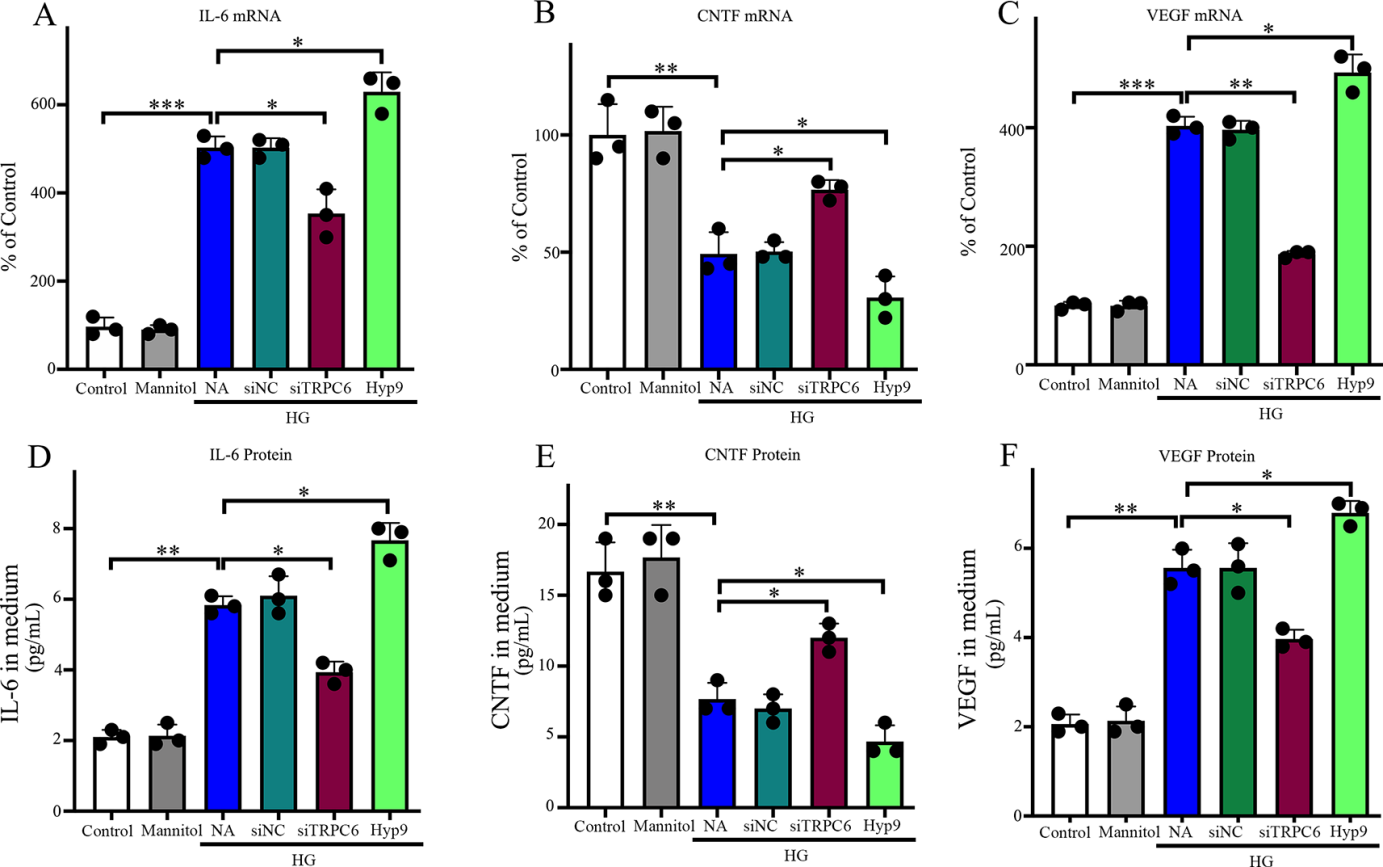
Effect of the TRPC6 channel on IL-6, CNTF, and VEGF expression and production in rMC-1 cells under normal glucose or HG conditions. The protein levels of IL-6 **(D)**, CNTF **(E)**, and VEGF **(F)** secreted by rMC-1 in the medium were quantified by ELISA. mRNA levels of IL-6 **(A)**, CNTF **(B)**, and VEGF **(C)** were determined by real-time PCR, and their values were standardized to β-actin mRNA levels in the same RNA sample. The data are expressed as mean ± SD; n = 3 for each group; *p < 0.05, **p < 0.01, ***p < 0.001.

We sought to examine the effects of TRPC6 knockdown on the mRNA expression and release of CNTF, IL-6, and VEGF in rMC-1 cells using an RNAi approach to reduce TRPC6 levels. HG treatment resulted in low mRNA expression and release of CNTF in or from rMC-1 cells (**Figures 7B** and **E**), but high levels of IL-6 (**Figures 7A** and **D**) and VEGF (**Figures 7C** and **F**). TRPC6 knockdown increased the mRNA expression and release of CNTF in or from rMC-1 cells and decreased the levels of IL-6 and VEGF. Exposure to 10 μM Hyp9 under HG conditions enhanced the effects of HG-induced changes (**Figure 7**).

## Discussion

Hyperglycemia is a major risk factor for various human diseases. Multiple studies have reported that the excitotoxicity caused by elevated glutamate in the extracellular space in experimental models of diabetes plays an important role in the pathophysiology of DR (Lieth et al., 1998; Lieth et al., 2000; Kowluru et al., 2001). Our results indicate Müller cells treated with 25 mM glucose exhibit decreased glutamate uptake activity because of decreased expression of the GLAST. The GLAST in Müller cells is mainly responsible for maintaining low synaptic glutamate levels in the retina. Significant decreases in glutamate transport mediated *via* the GLAST in Müller cells begins after just 4 weeks in diabetic rat models (Puro, 2002), which is consistent with reports showing significantly increased glutamate accumulation in diabetic rat retinas (Lieth et al., 1998; Lieth et al., 2000). Although the mechanism of dysfunction of the GLAST in Müller cells under HG remains unknown, Li et al. suggested that the dysfunction of GLAST is mainly caused by increased ROS levels (Li and Puro, 2002). In our study, inhibiting the generation of ROS under HG conditions by downregulating the TRPC6 channel enhanced the expression of the GLAST and improved the glutamate uptake activity of Müller cells under HG conditions. Some other studies showed that pro-inflammatory cytokines, such as IL-6 and TNFα, which trigger astrocyte activation, can cause a reduction in excitatory amino acid transporter (EAAT) expression. Activation of NFκB suppresses the transcription of EAATs. Loss of EAAT protein is associated with reduced glutamate uptake (Furman and Norris, 2014). In this study, we found that the level of IL-6 under HG condition decreased with TRPC6 knockdown. However, whether the NFκB pathway is involved in glutamate uptake is unknown and requires further research.

The Kir4.1 channel is the major inwardly rectifying channel in Müller cells and is widely thought to support K^+^ and glutamate uptake by Müller cells (Pannicke et al., 2006). Downregulation of the Kir4.1 channel when exposed to oxidative stress leads to an imbalance in K^+^ concentration, abnormal membrane depolarization, and subsequent swelling of Müller cells contributing to Müller cell dysfunction, resulting in gliosis and dysfunction of the GLAST, neuronal excitation, glutamate toxicity, and neuronal death (Francke et al., 2001; Pannicke et al., 2004; Olsen and Sontheimer, 2008). In our study, the Kir4.1 channel expression level decreased under HG conditions, GLAST expression levels decreased, and glutamate uptake was compromised simultaneously, which was consistent with previous studies.

The activity of Müller cells in response to oxidative stress may have cytoprotective properties in the early stages after damage and can be neuroprotective. However, this reaction can lead to a greater level of response described as gliosis, presenting as high levels of GFAP, which is detrimental to retinal tissue and exacerbates neuronal death, resulting in an increase in the retinal and vitreal levels of inflammatory factors, such as IL-6, while also decreasing levels of neuroprotective factors, such as CNTF (Bringmann et al., 2009). Some evidence indicates neuronal cell death is induced by the gliosis of Müller cells *via* the synthesis and secretion of inflammatory and neuroprotein factors. High IL-6 and decreased CNTF levels have been shown to contribute to retinal degeneration and neurodegeneration in DR (Bringmann et al., 2006; Bringmann et al., 2009). Furthermore, IL-6 has been found to be associated with vascular dysfunction and the promotion of angiogenesis, which suggest IL-6 may be a promising new therapeutic target to prevent diabetes-induced vascular damage (Rojas et al., 2010). In this study, we found the contents of CNTF secreted by Müller cells decreased under HG conditions. Knockdown of TRPC6 enhanced the secretion of CNTF and activation of TRPC6, while Hyp9 decreased the CNTF content. The opposite results were detected in the IL-6 content in the medium and expression of GFAP in Müller cells. The results showed that TRPC6 KD can prevent the gliosis of Müller cells and may provide a neuroprotective external environment for neurons in the retina.

Studies have suggested DR is a neurovascular disease of the retina, and the relationship between the excitotoxicity mediated by glutamate and the breakdown of the blood-retinal barrier (BRB) induced by VEGF is an interesting pathway linked to neurodegeneration with vascular impairment. Müller cells can produce many vasoactive growth factors. VEGF, a potent angiogenic and permeability growth factor, is known to be an important cause of BRB breakdown during the development and progression of retinal vascular diseases. It has been demonstrated that hyperglycemia induces an increase in extracellular glutamate levels in Müller cells, and subsequently, increased VEGF production and BRB breakdown was detected (Kusari et al., 2010; Shen et al., 2010). In this study, we have presented evidence that enhanced secretion of VEGF from Müller cells under HG conditions decreased with levels of extracellular glutamate, which were inhibited by silencing TRPC6. Application of Hyp9 increased the secretion of VEGF. Further study is warranted to establish whether TRPC6 KD can prevent BRB breakdown in DR.

Studies indicate Müller cells respond to oxidative stress and begin to die as DR progresses. Müller cells in the diabetic retina show roughly 15% cell death after 7 months (Feenstra et al., 2013). The death of Müller cells in the diabetic retina is associated with decreases in protective growth factors (Fu et al., 2015). Müller cells participate in the establishment of the BRB, which is comprised of the tight junctions between vascular endothelial cells and pericytes (Bringmann et al., 2006). The loss of Müller cells in diabetes has also been associated with aneurysm formation, a clinical characteristic of DR (Hori and Mukai, 1980). The consequences of Müller cell death promote the loss of retinal blood barrier integrity and increase vascular permeability. However, the mechanism by which Müller cells die is not clear. In this study, the decreased cell viability of Müller cells under HG conditions was inhibited by TRPC6 knockdown. The cell viability decreased after application of Hyp9, which was concentration dependent. The apoptosis of Müller cells under HG conditions was caspase 3-dependent, and silencing TRPC6 reduced cell death and the activity of caspase 3. These results indicate the TRPC6 channel is involved in Müller cell death induced by HG.

In summary, we described the high expression of TRPC6 in retinal Müller cells under high glucose conditions and downregulation of TRPC6 expression could enhance GLAST expression and improve glutamate uptake. Decreased intracellular Ca^2+^ concentrations caused by downregulating TRPC6 expression can decrease the production of ROS. Furthermore, silencing TRPC6 under HG conditions prevented the apoptosis of Müller cells and reduced the secretion of IL-6 and VEGF from Müller cells, while increasing CNTF expression. Our results suggest the TRPC6 channel may play a key role in the pathophysiology of DR, and downregulation of the channel may act as an antioxidative agent against neurovascular changes in retinal Müller cells in DR by decreasing intracellular Ca^2+^ concentrations. Thus, this evidence suggests TRPC6 may be a promising target for further research and therapeutic development in DR.

## Data Availability Statement

The raw data supporting the conclusions of this manuscript will be made available by the authors, without undue reservation, to any qualified researcher.

## Author Contributions

All authors listed have made substantial, direct, and intellectual contribution to the work and approved it for publication.

## Funding

This work was supported by the National Key R&D Program of China [2016YFC0904800 and 2019YFC0840607]; the National Science and Technology Major Project of China [2017ZX09304010]; the Program of the National Natural Science Foundation of China [Grant No.81770947, 81700842]; the Excellent Medical Young Talent Projects of Shanghai General Hospital (Grant No.06N1702019).

## Conflict of Interest

The authors declare that the research was conducted in the absence of any commercial or financial relationships that could be construed as a potential conflict of interest.

## Supplementary Material

Supplementary Figure 1 Effect of the siTRPC6 on TRPC6 expression in rMC-1 cells under normal glucose. The protein level of TRPC6 **(A)** was quantified by western blot. Densitometric analyse of TRPC6 protein levels was standardized against β-actin protein levels **(B)**. mRNA level of TRPC6 **(C)** was determined by real-time PCR, and the value was standardized to β-actin mRNA levels in the same RNA sample. The data are expressed as mean ± SD; n = 3 for each group; ***p < 0.001, vs control.
