# Supplementary figures and images for: High Glucose-Induced TRPC6 Channel Activation Decreases Glutamate Uptake in Rat Retinal Müller Cells
Source: Front Pharmacol. 2020 Feb 14;10:1668. doi: 10.3389/fphar.2019.01668 (PMC7033573; doi:10.3389/fphar.2019.01668)

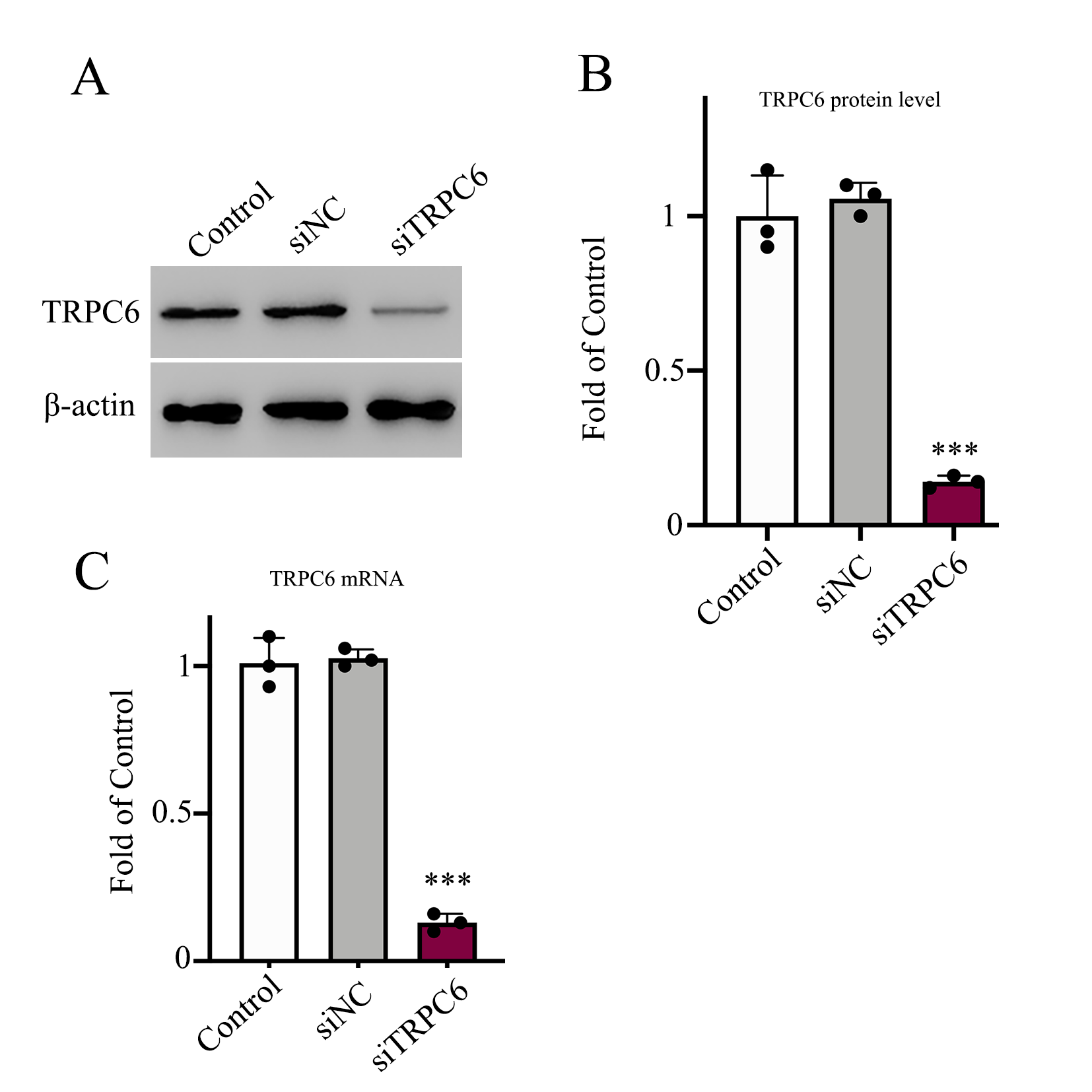

Supplement: Supplementary Figure 1 — Effect of the siTRPC6 on TRPC6 expression in rMC-1 cells under normal glucose. The protein level of TRPC6 (A) was quantified by western blot. Densitometric analyse of TRPC6 protein levels was standardized against β-actin protein levels (B). mRNA level of TRPC6 (C) was determined by real-time PCR, and the value was standardized to β-actin mRNA levels in the same RNA sample. The data are expressed as mean ± SD; n = 3 for each group; ***p < 0.001, vs control. [file Image_1.tif]
